# The Relationship Between Biological Noise and Its Application: Understanding System Failures and Suggesting a Method to Enhance Functionality Based on the Constrained Disorder Principle

**DOI:** 10.3390/biology14040349

**Published:** 2025-03-27

**Authors:** Yaron Ilan

**Affiliations:** Department of Medicine, Hadassah Medical Center, Faculty of Medicine, Hebrew University, P.O. Box 1200, Jerusalem 91120, Israel; ilan@hadassah.org.il

**Keywords:** digital health, variability, complexity, law of physics, artificial intelligence, biological noise

## Abstract

The Constrained Disorder Principle (CDP) offers a new framework for understanding how biological systems use and manage noise to maintain optimal functionality. This review explores the relationship between noise and biological systems at various scales, including genetic, cellular, and organ levels, and its implications for system malfunctions. According to the CDP, all systems require an optimal range of noise to function appropriately, and disease states can arise when these noise levels are disrupted. This review presents evidence supporting this principle across different biological contexts, such as genetic variability, cellular behavior, brain functions, human behavior, aging, evolution, and drug administration. For accurate clinical assessments, it is essential to distinguish between technical variability and intrinsic biological variability. When noise is adequately constrained, it serves as a fundamental mechanism for system adaptation and optimal functioning rather than simply a source of disruption. These findings have important implications for developing more effective therapeutic strategies and understanding biological systems’ dynamics. CDP-based second-generation artificial intelligence systems can help regulate noise levels to address malfunctions. These systems have improved clinical outcomes in various conditions by incorporating controlled randomness. Understanding these patterns of variability has significant implications for diagnosis, treatment monitoring, and the development of more effective therapeutic strategies across various medical conditions.

## Simple Summary

The Constrained Disorder Principle (CDP) helps us understand how biological systems manage noise to function well. This review looks at the connection between noise and biological systems at different levels, including genes, cells, and organs, and how this relationship can lead to problems when it goes wrong. The CDP suggests that all systems need some noise to work correctly. Health issues can occur when these noise levels change too much. This review shares evidence supporting the CDP in various biological areas, such as genetic diversity, cell behavior, brain functions, human behavior, aging, evolution, and medication effects. To make accurate clinical evaluations, it is crucial to tell the difference between technical and natural biological noise. When noise is kept within a proper range, it helps systems adapt and work optimally instead of just causing problems. These findings can lead to better treatment strategies and a deeper understanding of biological systems’ operations. Second-generation artificial intelligence systems based on the CDP can help manage noise and fix issues. Recognizing these patterns of variability is essential for diagnosis, tracking treatment, and creating more effective therapies for various medical problems.

## 1. Introduction

The Constrained Disorder Principle (CDP) defines all systems by their inherent variability and provides a platform for understanding systems’ malfunctions and using noise to overcome these challenges [1]. The CDP has recently undergone a thorough comparative analysis with various other theoretical frameworks that address the concept of variability. This examination highlights the CDP’s unique perspectives on how variability impacts development processes, contrasting with alternative theories that may emphasize different factors or mechanisms [2,3,4,5,6]. According to the CDP, the noise-based system enhances functionality and adaptability. The platform utilizes biological noise in a CDP-based approach to improve efficiency [4,5]. The following review aims to investigate the effects of noise on biological systems and its connection to malfunctions.

## 2. The Constrained Disorder Principle Platform

### 2.1. The Constrained Disorder Principle Provides a Platform for Using Noise

The CDP claims that noise is essential for the proper functioning of all systems in the universe [1]. It is a method for dynamic adaptation. The required range of noise for optimal performance varies based on internal and external system perturbations. This range of noise is described within dynamic boundaries, defining the noise level in a system [1].

The CDP is described schematically (not mathematically) by the formula B = F, where B represents the noise boundaries, and F represents the system’s functionality [7]. It suggests that systems can adapt to continuously changing environments by adjusting the noise level within dynamic boundaries. Per the CDP, disease states occur due to the malfunction of the noise boundaries, leading to an excess or lack of the necessary noise for proper functioning [8,9,10,11,12,13,14,15,16,17,18,19,20,21,22,23,24,25,26,27,28,29,30,31,32,33,34,35].

CDP-based second-generation artificial intelligence (AI) systems are designed to regulate the amount of noise in a system to overcome malfunctions. These platforms are based on the idea that dynamically increasing or decreasing the noise level in a system can help overcome malfunctions [36].

### 2.2. The Constructive Role of Noise: Diversifying Drug Administration Times and Dosages Creates a Random Environment and Helps Overcome Tolerance by Introducing Random Triggers to Cells and Biochemical Processes

The cellular response to drugs can vary significantly. The physiochemical factors influencing drug–target interactions within the cell’s microenvironment are not solely determined by simple diffusion and intrinsic chemical reactivity. The cell contains a non-uniform distribution of macromolecules that interact with medications in specific and nonspecific ways. Additionally, individual cells have varying abilities to sense their environment. Non-specific interactions can result in slow incorporation kinetics of DNA-binding and other binding sites [34].

Circadian rhythms affect the effectiveness of drugs. About 50% of human genes are estimated to follow a circadian cycle, and many are active in the liver. The inflammation, drug metabolism, and energy balance genes show cyclical activity. The internal circadian rhythms in human liver cells control the metabolism of drugs and inflammatory responses. Human liver cells show consistent oscillations, leading to changes in gene expression over time. Pro-inflammatory cytokines are induced circadian-dependent when stimulated by certain substances or infections. The timing of the circadian cycle significantly impacts the effectiveness and potential side effects of drugs [37].

Studies demonstrate how noise influences responses to drugs. Variability in HLA-DR-associated immunogenicity risk of biologics used for treating rheumatoid arthritis exists at individual and population levels. Determining this variability can help prescribe the most effective treatment for each patient with rheumatoid arthritis [38].

Drug tolerance poses a significant challenge in treating various diseases. The second-generation CDP-based AI system is designed to address this challenge by bringing treatment regimens closer to noise-based physiology, thus enabling the restoration of drug effectiveness [1,7,36,39,40,41,42,43,44]. This system diversifies drug administration times and dosages using random-based algorithms within approved ranges based on the drug’s pharmacokinetics.

By introducing regulated noise into the treatment regimen, the CDP-based AI system improved clinical and laboratory functions in patients with heart failure and diuretic resistance while reducing hospital admissions due to heart failure [7,45]. It stabilized disease progression in patients with multiple sclerosis. In patients with drug-resistant cancer, the noise-based system improved the clinical response to drugs, reducing side effects and improving clinical, laboratory, and radiological response rates [46]. A similar beneficial effect has been shown for patients with the genetic disorder Gaucher disease [33]. Future CDP-based systems implement personalized closed-loop platforms that continuously personalize treatment regimens based on individual variability [34].

### 2.3. Biological Noise from Genes to the Brain: The Correlation of Noise with Functionality and Malfunction

#### 2.3.1. Genetic Variability Is Associated with Functionality

According to the CDP, variability is inherent in all systems in the universe. The genetic data support the concept that genetic variability is necessary for adapting to internal and external disruptions.

Genetic variation is crucial for the correct development and adapting to changing environments. The continuous genetic selection reveals fine adaptation granularity while fluctuating environmental conditions are a common feature of natural habitats [47]. Natural populations adapt to changing selection pressures by adjusting their genetic design. Fluctuating selection at a fine scale helps maintain genetic diversity in populations and influences genome-wide diversity patterns at linked neutral sites, similar to genetic drift [48]. Analyzing genome-wide allele frequency data from a genetically diverse population of Drosophila over 12 generations showed that rapid, parallel changes in genomic variation occurred across replicates, driving adaptation during the population’s expansion, peak density, and collapse phases. Selection pressures varied within these ecological phases, and the standing genetic variation affecting fitness needed to be more apparent in static population sampling [48].

Genetic drift and random fluctuations in allele frequencies impact evolutionary outcomes when biological populations expand into new territories. Variability in the environment significantly affects competition between subpopulations. A population’s genetic composition reflects past range expansions, which are influenced by fluctuations in the growth of competing subpopulations. The variability from intrinsic demographic noise within a population and external random noise from a diverse environment impacts genealogical tree structure and genetic diversity. For substantial heterogeneity, the genetic makeup of the expansion front is mainly determined by the set of fastest paths through the environment. The statistics of these optimal paths then become the primary determinant of evolutionary dynamics, superseding those of the population’s intrinsic noise [49]. These data illustrate the CDP, where internal and external noises are managed using the inherent variability of biological systems.

The reasons behind temporary changes in adult characteristics are not entirely determined. Human mutation rates vary widely and are influenced by different mechanisms. This variation occurs within specific gene segments due to local DNA hypomethylation. The most significant differences in mutation rates are found within several kilobases of the transcription start site and extend downstream into the 5′ ends of gene bodies [50]. Analysis of population-level variance quantitative trait loci (vQTL) aligned with individual variability for several traits shows an overlap between trait variability assessed at the population or personal level. The genetic factors influencing the variability of specific anthropometric characteristics within an individual showed that non-directional variability had an SNP-based heritability of 2–5% for height, sitting height, body mass index (BMI), and weight [51].

It is essential to distinguish between technical noise and biological variation. Multiple modeling systems can accommodate genetic variability. Detecting changes in cell types using single-cell RNA sequencing (scRNA-seq) is complex due to variability between individuals and cohorts. Many current methods represent data variability as continuous hidden factors and perform clustering as a separate step or apply clustering directly to the data. These methods can sometimes make mistakes when accurately identifying different cell types. The standard method of identifying Highly Variable Genes (HVGs) can lead to distortion and bias in data analysis compared to actual biological variation. A feature selection model, Differentially Distributed Genes (DDGs), is a model that uses a binomial sampling process for each mRNA species to create a null model of technical variation, allowing for the accurate identification of real biological variation [51]. A computational tool, scDist, detects transcriptomic differences by replicating known immune cell relationships and minimizing false positives induced by individual and cohort variation [52]. A mixture model inference with discrete-coupled autoencoders (MMIDAS) is an unsupervised variational framework that learns discrete clusters and continuous cluster-specific variability. The model learns single-cell representations with robust definitions and interpretable, continuous within-cell-type variability. It identifies reproducible cell types and infers cell type-dependent continuous variability in unimodal and multimodal datasets [53,54]. One of the main challenges in spatially resolved transcriptomics (SRT) is evaluating the accuracy of computational methods. However, the currently available simulated SRT data are biased. “The cube” is a Python tool for simulating SRT data. It generates simulated data with varying spatial variability and allows for preserving spatial expression patterns of genes in reference-based simulations [55].

These examples support the role of noise at the genome level and the need to account for it in modeling.

#### 2.3.2. The Variability in Cell Structure Explains Its Functionality

Beyond the genome, the variability within and between individual cells holds unique information about their underlying functions.

Intrinsic cell variability and microenvironmental cues, such as cytokine and nutrient availability, influence the regulatory mechanisms guiding CD4 T cell differentiation. A model based on a regulatory network of early signaling events involved in cell activation and differentiation used a set of stochastic differential equations to assess the effect of noise intensity on differentiation efficiency and different effector phenotypes under defined conditions. The model shows how cytokines and nutrient availability affect T-cell differentiation, depending on the noise level. An increase in noise intensity decreases differentiation efficiencies. In a microenvironment of Th1-inducing cytokines and optimal nutrient conditions, altered noise levels reduced Th1 differentiation efficiencies, highlighting the network’s sensitivity to random variations. Further noise increments revealed that the network is relatively stable until very high noise levels of 20%, where the resulting cell phenotypes became heterogeneous. The network structure ensures the system reaches stable cell functionality despite varying biological intrinsic noise [56]. The data support the CDP by showing that noise levels affect functionality. While it does not specify the noise level needed for optimal performance, it indicates that efficiency declines beyond a certain noise threshold.

Cell signaling pathways drive cellular decision-making across different tissues and contexts. The transforming growth factor β (TGF-β) pathways, including the BMP/Smad pathway, are essential in determining cellular responses. Despite the pathway connectivity remaining nearly constant, the systems-level behavior of the Smad pathway varies from one context to another. Some cellular systems require rapid response, while others require high noise filtering. The BMP/Smad pathway balances trade-offs among three system-level behaviors, or “Performance Objectives (POs)”, which include noise amplification, response speed, and the sensitivity of pathway output to receptor input. By varying non-conserved parameters (NCPs), such as protein concentrations, the Smad pathway can be adjusted to emphasize any of the three POs. The concentration of nuclear phosphatase has the most significant effect on tuning the POs [57]. The data further suggest the need for noise regulation for proper functionality.

The microtubules undergo continuous shrinking and growth, a phenomenon known as dynamic instability, suggesting that proper stability in biological systems lies within their instability [58,59,60,61,62,63,64,65]. The variability in the elongation of spindles in C. elegans one-cell embryos can be measured by identifying three typical elongation patterns. These patterns explain over 95% of the differences between individual embryos in various conditions. Interestingly, the differences between genetically altered and normal embryos are similar [66].

The physiological variance in mitochondrial rRNA may play a role in the progression of metabolic syndrome. Differences in the mitochondrial rRNA sequence have been identified as a risk factor in the development of insulin resistance, which is associated with the accumulation of diacylglycerol induced by a reduction in the oxidative capacity of specific tissues. These metabolic disruptions result from variations in the 12S rRNA sequence, which affect mitochondrial ribosome assembly and translation [67].

The CDP accounts for the cell decision process, a fundamental developmental course at various scales of biological organization. Cellular decision-making involves cells taking on different, functionally important, and heritable fates even without any associated genetic or environmental differences. These stochastic cell-fate decisions create non-genetic cellular diversity, which can be crucial for metazoan development, optimized microbial resource utilization, and survival in a fluctuating and often stressful environment [68]. The microbiome constantly changes and shows daily fluctuations that can alter its composition. Even genetically identical populations growing in different locations on solid growth media exhibit significant variability in growth depending on their location. The causes of this variability change throughout population growth and can be attributed to the populations’ initial physiological states, the interactions between populations and their local environments, and the diffusion of nutrients and energy sources that connect these environments [18,69].

Oscillations are common in biological systems of all sizes. A synthetic oscillator called the “repressilator” in Escherichia coli was designed to be controlled using optogenetics to create the “optoscillator”. When bacterial colonies were exposed to periodic light pulses, the optoscillator behaved like a forced oscillator, displaying spatial ring patterns. This oscillatory circuit produced complex dynamics that resulted in distinct spatial patterns, revealing evidence of chaotic behavior [70].

Per the CDP, it is crucial to distinguish technical noise from inherent biological noise in algorithms to achieve results that closely resemble physiology. Electrically active cells, such as cardiomyocytes, exhibit size, shape, and electrical activity variability. Measurements of electrophysiological parameters, such as the midpoints of activation and inactivation of the cardiac fast sodium current, have shown variation in reported mean values between experiments and within experiments. The variability between experiments can be broken down into a correlated and an uncorrelated component, with the correlated component being more significant and affecting both midpoints almost equally. The observed variability can be attributed to biological and methodological issues, classified as factors within experiments or as correlated and uncorrelated factors between experiments [71].

Microbial growth variation is critical to Quantitative Microbiological Risk Assessment (QMRA). Modeling this variability is challenging, as the most common assumption is constant variability, implemented by an error term with constant variance added to the secondary growth model for the square root of the growth rate. This assumption contradicts microbial ecology principles, as differences in growth fitness among bacterial strains are more significant near the growth limits than at optimal growth conditions. Studies have shown that between-strain variability and experimental uncertainty are lowest at optimal growth conditions, increasing toward the growth limits. Experimental uncertainty increases towards the extremes, highlighting the need to analyze both sources of variance independently. Based on these findings, a secondary variability model has been designed to account for strain variability and environmental conditions [72]. A review of 16S amplicon sequencing studies in mice revealed that the time of sample collection can impact the findings of microbiome studies. This impact is more significant than daily experimental interventions or dietary changes. The point at which the microbiome composition differs between experimental and control groups varies in each experiment. Even sample collection times as close as four hours apart lead to vastly different conclusions [73].

In three-dimensional (3D) space-filling cell aggregates, cells undergo local topological transitions in the network of cell-cell interfaces, as described by the vertex model. A model reformulation, the Graph Vertex Model (GVM), involves storing the topology of the cell network into a knowledge graph with a specific data structure that allows for cell rearrangement events through simple graph transformations. Using a graph-database-management framework, a Python package implementing GVM has characterized an order–disorder transition in 3D cell aggregates. This transition is driven by active noise and shows that aggregates efficiently undergo ordering close to the transition point [71].

According to the CDP, variability can enhance a structure’s efficiency. Per the CDP, a noisy structure, as long as it is kept within the dynamic constraints, is fundamental for an optimal structure. Variability in the expression of critical ligands and receptors among different human pluripotent stem cell (hPSC) lines contributes to the variability in the success of producing cortical organoids. Individual organoids show high variability across different lines and experiments. Integrating organoids from various lines and donors can significantly enhance efficiency and growth [74].

According to the CDP, inherent variability acts as a mechanism for energy conservation, enabling systems to adapt more effectively to a noisy environment without expending excessive energy to combat it. Systems are constantly in a transitional state, known as the “edge of chaos”. Proper functioning of the dynamic boundaries of variability is crucial for this operation [4,65]. The theory of intelligent design predicts design patterns in nature, suggesting that certain aspects of nature can be better explained by intelligent agency rather than law-like regularities and chance events. Creational design patterns describe recurring patterns for building cell components. It includes templating, as cells must maintain the information in DNA and prevent it from being corrupted by time and chance. Structural design describes interconnections or relationships between objects in the cell, and behavioral design tells the behavior of cellular objects through time [75,76]. Many metabolites exist within cells, including energy storage forms like ATP. It is assumed that only an intelligent agent possesses these capabilities, which are inaccessible to random processes. Switching occurs when a continuum of input needs to be converted into a discrete output using ultrasensitivity or bistability [77]. Ultrasensitive switches enable a clear on/off response, ensuring that the system is either entirely in one state or the other rather than in an intermediate state. Positive feedback slows the response time, which is beneficial for multistage processes with delays and minimizes noise [68,78].

These examples suggest that biological noise must be considered when modeling cellular events while differentiating it from the technical noise related to the measurement.

#### 2.3.3. Heart Rate Variability Represents the Significance of Fluctuations Within Certain Limitations

Heart rate variability (HRV) is the heart’s beat-to-beat variation, reflecting its autonomic modulation. HRV analysis is a noninvasive method to assess autonomic function and provides insights into the dynamic interplay between sympathetic and parasympathetic influences on the cardiovascular (CV) system. It offers a noninvasive approach to studying the role of noise in biological systems [70].

The CDP concept emphasizes maintaining variability within specific boundaries for proper functionality. Conventional measures of HRV have shown modest associations with sudden cardiac death (SCD). High HRV is linked to reduced heart disease and cardiac event risk. Detrended fluctuation analysis (DFA) has revealed that using the ultra-short-term DFA2 α1 measured at rest independently predicts SCD [79]. Patients without atrial fibrillation had significantly lower HRV compared to those with atrial fibrillation. Reduced HRV was significantly linked to a lower risk of developing atrial fibrillation after surgery [80].

Per the CDP, the degree of variability can serve as a diagnostic biomarker. Decreased HRV in the neonatal period is linked to neurodevelopmental outcomes in preterm infants [81]. Systemic inflammation, induced by LPS, reduces HRV. Reduced HRV may be an early biomarker of neonatal sepsis [82]. Lower maternal HRV was linked to higher self-reported maternal depression and anxiety. Maternal HRV was also associated with elevated infant EEG theta–beta ratios [83]. Hemodynamic changes, as measured by HRV, in diabetic and nondiabetic patients undergoing laparoscopic cholecystectomy can guide preoperative anesthesia management for better perioperative outcomes [84]. The glomerular filtration rate is correlated with HRV in both the supine position and during active standing. Patients with end-stage renal disease exhibit changes in HRV dynamics during active standing, which are improved after hemodialysis and recovered after kidney transplantation [75]. Diffuse systemic sclerosis (dcSSc) differs from the limited lcSSc by a lower HRV in decubitus and orthostatic conditions [85].

The low-frequency to high-frequency (LF/HF) ratio of HRV parameters shows strong positive correlations with anthropometric parameters [86]. A meta-analysis of sixteen randomized controlled trials (RCTs) revealed that exercise training positively impacts HRV indices. It improves the standard deviation of normal-to-normal intervals (SDNN), the root mean square of successive differences in heart period series (RMSSD), and the absolute power of high-frequency band (HF) parameters. Exercise training enhances HRV parameters associated with vagal-related activity (RMSSD and HF) and sympathetic and parasympathetic activities (SDNN) [87]. HRV values are higher in elite badminton players, and higher HRV values lead to better agility performance [88].

Individuals with high HRV are more resilient to stress and consistently experience happiness [89]. HRV is linked to better memory of positive experiences. Higher HRV during non-rapid eye movement sleep (NREM SWS) and rapid eye movement sleep (REM) is connected to more remarkable improvement in the memory of pictures overnight [90]. HRV has been linked to resilience and emotion regulation (ER). Strong correlations exist between high resting HRV and HRV during tasks across all ER strategies. Individuals with high resting HRV showed lower levels of distress when using acceptance as an ER strategy. Whole-brain fMRI scans revealed that higher task HRV is associated with the activation of several brain areas [91]. A decrease in the parasympathetic parameter HF-HRV during the N3 sleep phase was observed [92]. HRV significantly decreases in the luteal phase of the menstrual cycle. Cyclical changes in HRV can serve as a biomarker of mood sensitivity to the menstrual cycle [93].

HRV is lower in patients with mental disorders, such as schizophrenia and bipolar disorder [94]. At six weeks postpartum, lower pregnancy HRV was linked with symptoms of depression or anxiety [95]. The brain regions that control HRV are also impacted by Alzheimer’s type dementia (AD) and Lewy body types (DLB). HRV was impaired in both types, with DLB showing more significant impairment in all HRV parameters than AD [96]. Autistic youth are predisposed to emotion dysregulation (ED). HRV objectively measures ED in these patients. Specific HRV profiles indicate long-term outcomes after receiving treatment [97].

These examples demonstrate that the range of biological variability can serve as a biomarker for health and disease.

#### 2.3.4. Blood Pressure Variability as a Biomarker for Disease

Blood pressure variability (BPV) is an example of how biological variability can be a biomarker. BPV is a significant risk factor in various disease states, including cerebrovascular and neurodegenerative diseases in older adults [98]. Increased BPV is associated with high CV risk in hemodialysis patients [99]. A study of over 8000 hypertensive participants followed for up to 21 years found that visit-to-visit variability in systolic blood pressure (SBP) is a significant predictor of CV outcomes. Mean SBP was a predictor of CV outcomes, and SBP variability (SBPV) was a predictor of CV events, even in participants with well-controlled BP [100].

SBPV is linked to outcomes in acute ischemic stroke. Remote ischemic conditioning (RIC) is effective in treating stroke and impact BP. Patients with acute moderate ischemic stroke who have higher SBPV benefit more from RIC in terms of outcomes [101]. Obstructive sleep apnea alters the balance between sympathetic and parasympathetic tone during sleep, leading to increased BPV at night. It enhances short-term and between-measurement BPV [102]. Generalized anxiety disorder is associated with increased SBPV [103]. Postpartum women with preeclampsia, with or without chronic hypertension, had lower HRV compared to normotensive postpartum controls. Higher BPV was observed only in those with preeclampsia not associated with chronic hypertension, suggesting that the autonomic profile of preeclampsia varies in patients with chronic hypertension [104].

A home BPV Risk Prediction Score can predict overall cardiovascular risk. This algorithm, including day-by-day home BPV, may assist in a blood pressure-centered approach to hypertension management [105]. The effectiveness of weight loss measures improved when CDP-based variability was introduced into vagal-based treatment [16,106]. A vagus nerve stimulation device worn around the neck sends gentle electrical pulses, which trigger the parasympathetic system to lower the heart rate and increase HRV [107,108].

The data support the concept that variability, as indicated by HRV and BPV, can guide treatment and that biological noise can serve as a biomarker to enhance diagnosis and therapeutic regimens [109].

#### 2.3.5. BMI Variability as an Independent Cardiovascular Risk Factor

The variability of BMI is calculated using the retrospective standard deviation (SD) and coefficient of variation based on multiple clinical BMI measurements leading up to the baseline. Increased BMI variability over time is linked to adverse CV events independently of traditional risk factors and mean BMI and is also associated with genetic variations. In a study involving 157,410 individuals, BMI variability was linked to adverse CV events, regardless of the overall mean BMI and genetic risk score. The coefficient of variation of BMI was associated with a 16% higher risk for composite cardiovascular disease across all groups [110]. Higher BMI variability was linked to an increased risk of being hospitalized due to COVID-19 [111].

#### 2.3.6. Altered Gait Variability Can Serve as a Biomarker for Disease

The variability in the walking pattern can be measured by looking at the time intervals between each stride [112]. Nonlinear variability during steady-state gait indicates walking adaptability and is a health signature. A study on healthy young adults showed that performing a secondary task while walking, such as using a smartphone, affected the average and variation in stride intervals. The decreased consistency during the cognitive task indicated a decline in the healthy walking pattern due to the added mental and physical demands [113].

Lower bone mineral density is linked to increased variability in walking patterns, particularly at the lumbar spine. The higher gait variability was associated with the deterioration of mobility-related brain areas. The reduced bone mineral density in the lumbar spine contributes to the higher variability in walking patterns by impacting the sensorimotor cortex and the primary motor cortex [114].

Juggling is a complex activity that requires motor, visual, and coordination skills. Learning to juggle can lead to changes in the brain’s structure, specifically in the area at the back of the brain known as the posterior intraparietal sulcus. This area becomes more developed due to juggling, and it is involved in adjusting our awareness of the space around us by moving attention between the left and right positions of the juggling balls [115]. Skilled swimmers use a specific movement pattern called flutter kicking that creates a wave-like motion, moving from the head to the feet with a consistent or increasing speed. It is essential for this movement to have minimal biological noise and to be executed with low variability [116].

These examples illustrate the connection between biological variability, brain structure, and behavior, supporting the CDP-based idea that variability is a fundamental biological process impacting assembly and functionality.

#### 2.3.7. The Brain Function Is Marked by Variability

Per the CDP, cognitive development and brain function mandate a degree of noise to ensure plasticity and adaptability [28,31].

The brain processes information consistently despite its inherent activity inconsistency. When the brain is repeatedly exposed to the same stimuli, its neural responses become more consistent, improving perceptual consistency. This input-dependent consistency, selective consistency, is achieved through self-organization and plasticity within the neural system. The acquisition of selective consistency is independent of performance optimization. The plastic neural network achieved selective consistency when operating near the edge of chaos—a critical state maintaining a delicate balance between order and chaos. It suggests that a brain near criticality can self-organize its structure with plasticity to improve perceptual consistency [117].

Neural activity in the cortex displays a wide range of firing variability and correlation structures. The correlated neural variability impacts the quality of neural codes, either positively or negatively. When spiking neurons are involved, computing covariance provides a unified perspective on neural representation and computation amid correlated noise. Nonlinear coupling of correlated neural variability can be performed by constructing neural network models for performing covariance-based perceptual tasks. The brain extracts perceptual information from noisy sensory stimuli to create a stable perceptual whole, highlighting correlated variability’s role in cortical information processing [118].

The strength of connections between individual neurons can vary due to differences in the likelihood of releasing neurotransmitters in response to action potentials. The short-term plasticity of these synapses varies based on the type of motor neuron and the muscle it connects to. These synapses are randomly distributed within the nerve terminal of the motor neuron and exhibit diverse firing frequencies and responses to changes in frequency. Despite these differences, the overall distribution of neurotransmitter release remains stable due to a balance between facilitating and depressing synapses and their strength variations. This stability ensures consistent neuronal function across different behavioral conditions [119].

The data illustrate the CDP and emphasize the concept of order from disorder in biological systems, where living on the edge of chaos enhances functionality. Biological systems improve functionality by embracing disorder to adapt to perturbations [4,5,120,121,122,123].

Functional connectivity describes the correlated variability across neurons and brain regions, and both correlated variability and its modulation by attention depend on the specific types of anatomical connections between neurons. Visual cortical neurons exhibit variability in their responses when presented with the same stimulus multiple times, and some of this variability is consistent across neurons. Attention can improve visual perception by decreasing this shared variability in spiking activity. However, the presence and modulation of shared variability due to attention vary within or between different brain areas and depend on numerous factors, such as the type of neurons involved. When attention was directed, neurons connected through excitatory synapses showed a substantial decrease in spike count correlations. Attention diminishes shared variability in excitatory circuits, even when the effects of attention on randomly selected neurons within the same area are minimal [124].

An analysis of eye movement data collected during a controlled task shows that measures of randomness in eye movements, such as gaze transition entropy and stationary gaze entropy, decrease when operators need to continuously monitor a compensatory tracking task that involves dynamic operational activities. Gaze transition entropy provides insight into how operators allocate their attention when performing these tasks consistently [125].

The spatial distribution of individual variability in the whole-brain functional network architecture of the auditory cortex shows an inherent hierarchical axis of variability extending from the medial to lateral orientation, with the left auditory cortex showing more pronounced variations. This variability correlates with variations in structural and functional metrics in the auditory cortex. The findings highlight the role of individual variability in functional connectivity in cortical functional organization and its association with functional specialization from the perspectives of activation, connectome, and cognition [126]. Cerebrovascular Reactivity (CVR) is the brain’s vascular response to a vasodilatory stimulus. It is measured using fMRI during breathing challenges that change arterial CO_2_ levels. The relationship between resting-state fluctuations metrics and CVR across different sessions demonstrated high intra-individual variability, suggesting a marked underlying variability in brain function [127]. The data further support the association of structure with variability.

Neurons predict and actively influence their future inputs through their outputs. Neurons, especially those beyond early sensory areas, steer their environment toward a specific desired state through their outputs. It is challenging to model neurons as feedback controllers of their environments when the dynamical system characterizes the environment. The environment comprises both synaptically interlinked neurons and external motor sensory feedback loops, enabling neurons to evaluate the effectiveness of their control via synaptic feedback. A model designed to characterize the operational variability and noise in the brain showed the shift from potentiation to depression in spike-timing-dependent plasticity with its asymmetry and the imprecision in spike generation under constant stimulation [128]. The findings correspond with the CDP, which outlines the interactions between dynamic environments and systems. It necessitates the continuous adaptation of variability within a system to better adjust to its environment.

#### 2.3.8. Variability in Behavior Offers an Advantage for Better Adaptation to Noisy Environments

The CDP also accounts for behaviors implying that randomness within constraints can improve outcomes.

Animals, like humans, often encounter situations where information is incomplete or unreliable. To thrive in a changing and uncertain world, animals benefit from making educated guesses about hidden aspects of their environment to make decisions and plan future actions. Some examples include finding a food source based on uncertain smells, predicting the movement of a target while chasing it, or designing efficient routes through multiple destinations. Various strategies exist for making and using these educated guesses to guide behavior. One common approach is to find the best plan for maximizing performance on a specific task. Focusing solely on one optimal strategy overlooks various practical techniques that could ensure satisfactory performance [129]. Homing refers to an animal’s ability to navigate and return to a specific location after traveling long distances and unfamiliar terrain. Animals exhibit some level of randomness in their navigation, but beyond a certain point, this randomness does not affect the homing duration. Computer simulations with robots that mimic animals indicate that reorientation and randomness work together to help these animals return to their homes efficiently [130]. Humans often use quick decision-making methods, sometimes deviating from the theoretically optimal paths, showing significant variability and flexibility in their behavior within and across situations [129]. These data align with the CDP by using random selection to address uncertainty better.

In traditional approaches, the best task strategy is determined using Bayesian techniques, which offer a mathematical model for updating beliefs based on new information, rather than focusing only on the best strategy, a wide range of potential strategies can be identified, spanning from nearly optimal to suboptimal ones. It involves creating ‘small programs’ with a limited number of internal states, which are memory pockets that store information about past actions and outcomes, thus limiting the complexity of the strategy. Even with a few memory states, animals could perform well using diverse strategies tailored to specific situations. A tree embedding algorithm revealed a ‘smooth’ nature, where small changes in structure led to small changes in behavior, showing rough spots where specific mutations caused significant shifts in behavior and performance. These findings align with real-world learning, which often involves incremental gains punctuated by occasional leaps in understanding. The ‘sloppiness’ in animal strategies refers to the idea that multiple strategies can achieve similar outcomes, even if they operate differently. It suggests variations in the strategies and their underlying neural implementations [129].

The concept aligns with the CDP, which suggests that variability is a platform for improved efficiency, allowing systems to adapt to changing scenarios and operate effectively in unpredictable environments. The CDP also offers a framework for overcoming obstacles. However, it does not consider the role of learning, which implies a form of memory within the system. According to this principle, systems are designed to function under dynamic conditions and continuously adjust their noise levels in response to perturbations. In the context of the CDP, “memory” is embedded in the boundaries of variability, but it does not equate to learning [4,5].

Variability is a fundamental aspect of cognition, which may provide new insights into developmental processes. Performance on cognitive tasks fluctuates, and the same individual completing the task differs in their responses from moment to moment. Cognitive fluctuations have been implicitly ignored and treated as measurement errors, focusing on aggregate measures such as mean performance. High interindividual differences in cognitive variability were demonstrated across numerous cognitive tasks with millions of trials [130]. The leading neurobiological hypotheses regarding autism spectrum disorder suggest increased variability, underconnectivity between brain regions, and atypical amygdala function [131]. Patients with ADHD exhibit distinct impairments in various aspects of cognitive control across multiple tasks and performance measures. They display higher response variability and poorer interference control, which underlie the pathophysiology of ADHD [132].

According to the CDP, using “mean” is insufficient because variability characterizes the behavior and acts as a mechanism for improved performance. It implies that inappropriate brain and behavior variabilities are associated with diseases. These changes indicate a malfunction in the variability boundaries, causing an inappropriate increase or decrease in the variability range, which leads to a diseased state [4,5].

Robots could improve their ability to handle intricate tasks such as search and rescue operations or space exploration missions by emulating the strategic adaptability observed in animals. Understanding the principles of learning and adaptation lays the groundwork for technologies capable of thriving and adjusting in complex, real-world scenarios [129]. Goal-directed navigation involves continuously integrating uncertain self-motion and landmark cues to develop an internal sense of location and direction. A computational model was developed to describe probabilistic path planning under uncertainty within the optimal feedback control framework. It explains the diverse human navigational strategies, previously thought to be distinct behaviors, and predicts the errors and variability of navigation across numerous experiments. It clarifies how sequential egocentric landmark observations create an uncertain allocentric cognitive map for route planning and movement execution [133]. The Color Spin Wheel is a digital tool that assists decision-making by adding randomness to the process. It makes decision-making more fun and accessible, helping to reduce decision fatigue. Introducing an element of randomness relieves the pressure of making choices and adds an interactive aspect to the experience [134].

These examples illustrate how using the CDP-based approach to manage variability improves functionality.

#### 2.3.9. Aging Is Associated with Altered Variability Outside of the Normal Range

The CDP defines aging as a malfunction of the variability boundaries, leading to an inappropriate degree of variability in a system. It offers the option of regulating variability to overcome some aging challenges [26,135].

Random and non-random processes influence biological aging. Age-related signals, such as genetic and epigenetic changes, are random. Some of these changes are harmless but impact cellular function over time, causing stress [136,137,138,139]. Likewise, BPV increases gradually as a person ages [140]. The damage caused by metabolic processes reflects the increase in entropy and is referred to as the entropic component of aging, the accumulation of damage due to normal metabolic processes. Over time, the increasing damage leads to pathology [141,142,143,144]. In humans, the dynamic component that drives aging becomes dominant during the last couple of decades of life. Interventions affecting this component without stopping the accumulation of entropic damage may slow aging [142,145].

DNA methylation clocks can estimate chronological age and, to some extent, biological age. Even random variation in simulated data can be used to create aging clocks. The process through which age-related DNA methylation changes occur is somewhat random and accounts for up to 75% of the accuracy of Horvath’s clock. This percentage increases to 90% for more accurate clocks but is lower for the PhenoAge clock [146]. Aging clocks measure how effective interventions and treatments are in slowing aging and preventing age-related diseases. These clocks can account for the variation in DNA methylation or transcriptomic data, and their accuracy may be due to a programmed process that underlies aging [147].

Maintaining and removing information in the mind are two critical cognitive processes that decline with age. A combination of beta-band neural oscillations, which regulate working memory, and cross-trial neural variability, a feature of brain dynamics, govern adaptive cognitive processes. A decrease in beta variability during information maintenance predicted memory performance in younger adults but not older ones, while post-response beta variability correlated with memory performance in older adults [148].

In aging individuals, changes in neural variability may provide unique information about their emotional experiences, which can vary from person to person. The variability of neural activity from moment to moment is essential for learning and adaptability in behavior. This variability has been associated with differences in task performance and brain activity as people age. Changes in cortical neural variability during natural experiences reflect changes in specific brain functions, separate from resting-state variability and average neural activity. In older adults, there is a shift in neural variability in the medial and lateral orbitofrontal cortex during emotional experiences. This shift is linked to increased uncertainty in processing changing emotional experiences in older adults [149].

The data support the CDP concept that the variability range is related to aging and can be a therapeutic target for slowing this process.

### 2.4. Variability in Tests: Distinguishing Technical Noise from Biological Noise

According to the CDP, variability is a characteristic of any system. This inherent variability is mandatory for proper functioning. In contrast, technical variability refers to an inter-laboratory variability when measuring systems and to the intra- and inter-subject variabilities during these measurements. Technical noise must be distinguished from intrinsic noise in systems [42,109].

The responsiveness of platelets to clopidogrel varies over time in many patients, impacting clinical outcomes. When each patient was evaluated individually, it was found that 15% of patients taking 75 mg of clopidogrel and 11% of patients taking 150 mg experienced a change in their responder status when tested at two different time points. Additionally, more than 40% of patients showed a shift in platelet reactivity when tested multiple times [150]. The decline in Forced Expiratory Volume in one second (ppFEV1) indicates the progression of Cystic Fibrosis (CF). Increased ppFEV1 variability is a relative increase or decrease of at least 10% from a two-year averaged baseline. Survival curves showed a higher lung transplant/mortality risk as the cumulative ppFEV1 variability increased in CF [151]. Increased and decreased serum uric acid (SUA) levels were associated with smaller brain white matter volume. Higher SUA levels are linked with widespread disruptions in brain microstructural integrity, including lower global fractional anisotropy and higher mean and radial diffusivity. Elevated SUA is linked to cognitive decline. Changes in SUA levels, rather than the specific direction of change, play a significant role in brain health [152].

These examples underscore the need to consider technical and biological variability before making clinical decisions and the significance of changes in human pathology.

### 2.5. Models Must Consider the Variability Inherent in Biological Systems

Per the CDP, noise can functionally facilitate transitions between stable states in various stochastic dynamics and, therefore, needs to be accounted for in modeling [2].

It is challenging to model real-world problems, which often involve randomness and unpredictability. This wide-ranging diversity in systems can cause researchers to overestimate the significance of their findings, leading to uncertainty in measurements affecting the insights gained from experiments. Variations can span a broad spectrum, from mild to extreme [153]. Noise is typically problematic when extracting valuable patterns from time series data. As a result, traditional methods focus on learning patterns while minimizing the impact of noise [154]. Introducing randomness into specific algorithms can make solving them more efficient and faster. It helps to clarify the relationship between different types of problems in terms of their difficulty and the role of randomness [155]. Incorporating randomness was a method used to create faster algorithms for a wide range of applications [156]. Probabilistic algorithms can be made deterministic by removing randomness, revealing the link between computational complexity and randomness [157].

Randomness is a crucial extension of the original Turing Machine. A computer can simulate it by computing deterministic but random-looking or “pseudorandom” numbers [155]. AI relies on both massive parallelism and randomness. The stochastic gradient descent algorithm can train most neural nets, and the ‘temperature’ setting is used in nearly all chatbots to introduce a level of randomness into their output [158,159]. Noise in both natural and engineering systems can have a constructive impact. Noise-induced stochastic resonances can enhance system performance. In an alternating current-driven liquid-crystal electroconvection system, a transition between these resonances was observed using a combination of amplitude and phase noises. The internal material properties and external noise parameters play a role in controlling this transition. By managing this transition between resonances, specific outcomes for desirable or undesirable system performance can be achieved [160].

Large-scale multimodal neural recordings often suffer from significant noise, making extracting meaningful information and analyzing the data difficult. Denoising is a computational tool designed to adaptively adjust thresholds based on signal characteristics and noise levels, effectively separating signal and noise components without needing specific data transformations. When applied to large-scale neural recordings from mice’s hippocampal and olfactory bulb networks, denoising improves the signal-to-noise ratio (SNR) and spike firing patterns [161]. However, according to the CDP, it may eliminate both technical and inherent noise, thus reducing the accuracy of the results.

Biological systems exhibit complex dynamics that can often be accurately represented using differential equations and machine learning methods, offering an alternative approach to model construction. The General Linear Model (GLM) is commonly used, where the error term is typically considered noise. However, the target variables may have inherent variances in biological systems. A modified GLM has been proposed to model biological variance and non-biological noise explicitly. This modified model, EM for Separating Variances (EMSEV), uses the Expectation and Maximization (EM) scheme to differentiate biological variance from noise. EMSEV calculates targets and noise variances, enabling a statistical analysis by comparing two distributions. The discrepancy between EMSEV outputs and the pre-defined distribution parameters increased with the noise level. When the noise magnitude and the variance of the target variables were similar, there were 3% and 10–16% deviations in the estimated mean and covariance of the target variables, respectively, along with a 1.7% deviation in noise estimation [162].

Differential equation models can be learned from data through model discovery using sparse identification of nonlinear dynamics (SINDy). SINDy encounters difficulties with realistic levels of biological noise. A data-driven model, discovery, and selection framework using hybrid dynamical systems was developed where neural networks approximate the unknown dynamics of a system, allowing for denoising the data while simultaneously learning the latent dynamics. It permits the correct inference of models even in high levels of different types of biological noise, providing a practical framework for cases where data are noisy and sparse and where the underlying biological mechanisms are partially known but not fully understood [163].

The movement of molecules and proteins inside biological systems appears random. Nature has harnessed this randomness to perform valuable functions, such as driving the immune response and regulating protein folding and unfolding. An analog computer can decode nature’s randomness and determine unpredictable processes, such as the movement of molecules and proteins in biological systems. Levitating the particles creates a clean slate, as they are not in contact with their environment. Electric fields then replicate realistic natural scenarios, with control signals used to generate random forces [164].

A mathematical model was developed to assess the effect of treatment on the dynamics of HIV/AIDS and pneumonia (H/A-P) co-infection. The model showed that random perturbations in H/A-P co-infection play a role in controlling the spread of the epidemic. The amount of infection eliminated is closely correlated with random perturbations, and better outcomes were obtained when analyzing facts using random perturbations [165].

Human-induced pluripotent stem cell-derived cardiomyocytes (iPSC-CMs) can be created from healthy and diseased subjects and renew themselves. However, they display significant variations in their characteristics. Models of iPSC-CM electrophysiology predict cell responses but do not account for the variability observed in these cells. A computational method has been created to simultaneously estimate multiple model parameters, which produce personalized models, leading to better predictions of how cells respond to drugs and uncovering specific differences in their electrical properties that contribute to variations in cardiac maturity and susceptibility to arrhythmias. The genetic algorithm includes a heuristic parameter calibration method that adjusts ion channel parameters in a mathematical model of iPSC-CM physiology [166].

Inferring dynamical models from omic data is significant due to the stochastic nature of biological processes. In omics, statistically independent cross-sectional samples are expected to be present at a few time points to infer the underlying diffusion process that generated the data. Existing inference approaches simplify or ignore the intrinsic noise in the system, compromising accuracy. The probability flow inference (PFI) approach infers the phase-space probability flow that shares identical time-dependent marginal distributions as the underlying stochastic process. It disentangles force from intrinsic stochasticity while retaining the ease of ODE inference. PFI enables accurate parameter and force estimation in high-dimensional stochastic reaction networks, allowing the inference of cell differentiation dynamics with molecular noise [167].

The examples highlight the need to differentiate between inherent biological noise and technical noise. Acknowledging the randomness in modeling biological systems is essential for improving their accuracy.

### 2.6. A Role for Randomness in Evolution Processes

According to the CDP, evolution is not merely about improvement; it involves adapting to dynamic and noisy conditions [6].

Darwin may have been correct in stating that it is futile to ponder the origin of life, as life may not have a single origin but may have emerged from various sources [168]. Even bacteria, the simplest life forms, have evolved through numerous successive steps. The most crucial steps may have been significant and sudden rather than Darwin’s gradual, everyday mutation and selection. These “major evolutionary transitions” involve simpler, less complex replicating entities becoming interdependent to create a larger, more intricate, and more capable replicator [169]. When a byte is changed, it is usually due to random mutation, and even if a valid instruction causes the change, it is arbitrary and purposeless. However, after a few million interactions, the tapes start to reproduce, creating copies of themselves and each other, and randomness transforms into complex order [170,171].

According to the CDP, evolutionary processes do not follow a trajectory of improvement; they are dynamic processes that adapt to perturbations by regulating the range of variability in systems [5].

An extension of thermodynamics that deals with the statistical behavior of matter undergoing random thermal fluctuations above absolute zero indicates that randomness influences everything. According to the second law of thermodynamics, entropy increases over time in a closed system [172]. Life may appear to defy the second law of thermodynamics. Living organisms persist, grow, and become more intricate over time instead of deteriorating. It does not violate the laws of thermodynamics because life cannot exist in a closed system; it needs an input of free energy [173]. The complexity might not stem from simple Darwinian selection acting on the random structures generated over a hypothetical million generations. Instead, it could emerge without any random mutations, relying solely on the initial randomness. The result resembles a nonsensical text, which is far from what a million generations could create and is too limited to contain more than a few consecutive characters of functional code [174].

The CDP accounts for evolutionary processes. Random mutations are considered part of the ongoing dynamic variability and are the primary source of genetic variation that underpins evolution [4,5,6]. These concepts align with the CDP “order from disorder” notion, a universal law underlying all systems [4,60,61,62,63,64,65,120,121,122,123,175,176,177,178]. According to the CDP, there is no set path for evolution, and systems are designed to operate in dynamic environments, making the inherent randomness a fundamental process underpinning development and functionality.

The concept of symbiosis plays a central role in evolution, going beyond just random mutation and selection. Before tapes begin replicating, there is a gradual increase in computation. Imperfect replicators, minor bits of code with some chance of generating more code, emerge rapidly. According to this hypothesis, there is a selection process in which code leads to the creation of more code. This self-catalyzing process is more important than random mutation in generating novelty. A symbiotic event occurs when proliferating bits of code combine to form a replicator [179]. Per the CDP, the process is always random, and there is no “first template” based on which it starts. Instead, it is a continuous dynamic process of adapting to randomness and perturbations.

Robert Sapolsky suggests that the feeling of being a free agent is primarily an illusion created by biology and its interaction with the environment. Making a decision and knowing that one could have chosen differently is typically enough to determine that a freely made choice has occurred. According to Sapolsky, there is no point at which a neuron, brain region, or person can act independently of preceding events [180]. This concept aligns with the CDP, where “free will” is embedded within the dynamic boundaries of variability [34].

The relationship between species’ competitiveness and traits’ variability within the same species may allow different species to coexist in communities of young plants. When less competitive species have higher variability in their traits compared to their more competitive counterparts, the coexistence of these species can be promoted. No relationship between the variability of characteristics within the same species and the competitiveness of the species was found when they were grown in environments without competition and with uniform conditions. However, an inverse relationship emerged when different species competed, and the environment was varied. The relationship was influenced by the plastic response of the less competitive species. Less competitive species showed a more significant increase in trait variability, especially in traits related to fine roots, in response to competition, varied environments, or both [181]. Despite their inherent variabilities and dynamics, living organisms establish strong forms and functions through interactions between molecules, cells, and tissues. While molecular and cellular variability is often considered a disadvantage, it is crucial in cellular decision-making and robustness [182].

Early mammalian embryos show variability in gene expression, mechanical properties, and the timing of cell divisions. Despite these random dynamics, the embryos develop robust forms and functions. Temporal variability and cell mechanics play a significant role in the robustness of embryonic development. During the eight-cell stage, cell desynchronization leads to increased stochastic behavior and cell autonomy. Meanwhile, spatial variability between embryos decreases due to compaction and topological transitions toward specific configurations. It supports that temporal variability is essential for robust morphogenesis [182]. One of the two X-chromosomes in female mammals is epigenetically silenced in embryonic stem cells through X-chromosome inactivation. The proportion of inactivated parental alleles, the X-chromosome inactivation ratio, varies widely among individuals, making it the most significant example of epigenetic variability within mammalian populations. The randomness of X-chromosome inactivation during embryonic development explains the variability in X-chromosome inactivation among mammalian populations, with genetic factors playing a minor role [183].

These data support the CDP’s idea that inherent variability is essential for functionality. It demonstrates that variability is fundamental to all processes of development and existence. Per the CDP, there is no free will in choosing what to do but no determinism in its simple meaning. Responses are dictated by randomness within dynamic boundaries, which determine the range of noise in a system [34]. It implies that if all biological responses are dictated by randomness within constraints, then this can be viewed as a type of determinism [34].

## 3. Conclusions

Variability is often associated with uncertainty and viewed negatively; however, studies highlight its essential role in biological systems. According to the CDP, a certain level of variability is crucial for adequately functioning these systems. A noise-based approach can enhance both functionality and adaptability. The CDP provides insights into system malfunctions and demonstrates how noise can help address these issues. Recent data emphasize distinguishing between technical noise and biological variability and considering biological noise when modeling systems to improve accuracy. Future research employing CDP-based algorithms considering biological variability is essential for generating outputs accurately representing normal physiological conditions. These studies will enhance our understanding of complex biological systems by integrating diverse biological data and accounting for variations across different populations. Moreover, this approach will help assess the relevance of findings across multiple systems, leading to more practical applications in fields such as personalized medicine, drug development, and disease modeling.

## Data Availability

All data are available on public resources.

## Abbreviations

CDP: Constrained Disorder Principle; CV: cardiovascular; AI: artificial intelligence; HRV: heart rate variability; ANS: autonomic nervous system; BPV: blood pressure variability; SBPV: systolic blood pressure variability.
